# Down-regulation of PRR11 affects the proliferation, migration and invasion of osteosarcoma by inhibiting the Wnt/β-catenin pathway

**DOI:** 10.7150/jca.62491

**Published:** 2021-09-13

**Authors:** Ke Li, Hongtao Yu, Chunbing Zhao, Jing Li, Rui Tan, Lei Chen

**Affiliations:** 1 Orthopedic Center, First Affiliated Hospital, School of Medicine, Shihezi University, No.107 North 2nd Road, Shihezi, Xinjiang 832008, P. R. China.; 2 Department of Pharmacy, First Affiliated Hospital, School of Medicine, Shihezi University, No.107 North 2nd Road, Shihezi, Xinjiang 832008, P. R. China.

**Keywords:** PRR11, osteosarcoma, Wnt/β-catenin pathway, Epithelial-mesenchymal transition

## Abstract

**Purpose:** This study aim to explore the effect of down-regulation of PRR11 (proline-rich protein 11) on the proliferation, invasion, migration, Wnt/β-catenin signaling pathway and EMT of osteosarcoma cells.

**Methods:** Immunohistochemical staining, fluorescent quantitative PCR and western blotting were used to detect the expression level of PRR11 in osteosarcoma tissues and osteosarcoma cells. After SiRNA down-regulated the expression level of PRR11, CCK8 was used to detect cell proliferation ability, Transwell chamber to detect cell invasion ability, scratch test to detect cell migration ability, and flow cytometry to detect cell apoptosis. Western blotting was used to detect the expression levels of wnt/β-catenin pathway related proteins and key epithelial-mesenchymal transition proteins.

**Results:** PRR11 is highly expressed in osteosarcoma tissues, and its expression level is related to tumor size, Enneking stage of tumor, lymph node metastasis and patient prognosis. The low expression of PRR11 can inhibit the proliferation, migration and invasion of osteosarcoma cells, and promote apoptosis. Down-regulating the expression of PRR11 will inhibit the expression of Wnt pathway related proteins β-catenin and p-GSK-3β, enhance the expression of p-β-catenin, GSK-3β, and increase the expression of downstream genes CyclinD1 and c-Myc in the Wnt pathway. At the same time, the expression of PRR11 was down-regulated, the epithelial marker E-cadherin was significantly increased, and the expression levels of mesenchymal markers Vimentin and Fibronectin were significantly reduced.

**Conclusion:** Down-regulation of PRR11 can inhibit the proliferation, migration and invasion of osteosarcoma cells, and its mechanism may be related to down-regulation of PRR11 to inhibit the Wnt/β-catenin signaling pathway and thus prevent the EMT process. Therefore, PRR11 may be used as an oncogene to promote the occurrence and development of osteosarcoma, and is a potential prognostic indicator and therapeutic target in osteosarcoma.

## Introduction

Osteosarcoma is one of the most common malignant tumors in children and adolescents, with poor prognosis and high mortality 1. According to domestic and foreign data, the 5-year survival rate of patients with osteosarcoma has been significantly improved by 51%-75% 2. However, the 10-year survival rate and long-term tumor-free survival rate of patients with osteosarcoma are still less than 50%, mainly due to lung metastasis and high local recurrence rate 3. Therefore, exploring new diagnostic molecular markers and clinical treatment targets is of great significance for the early diagnosis and treatment of osteosarcoma and improving the prognosis of patients.

PRR11 is a proline-rich protein, which is encoded by the PRR11 gene. The PRR11 gene is located in the 17q23 amplification region. The copy region of 17q23 is significantly increased in brain tumors, lung cancer, breast cancer and ovarian cancer 4. PRR11 is highly expressed in malignant tumors such as pancreatic cancer 5, hilar cholangiocarcinoma 6, and ovarian cancer 7. However, the mechanism of PRR11 in osteosarcoma is still unclear.

## Materials and methods

### Tissue specimens

Osteosarcoma tissues that were surgically resected at the First Affiliated Hospital of Shihezi University between January 2015 and January 2020 were collected. After removing the tissue sample, put it in liquid nitrogen and freeze it, and then store it at -80 °C. The study included 62 paraffin-embedded, post-operatively confirmed specimens, matched with 62 paracancerous tissue specimens (>2 cm away from the tumour edge, pathologically confirmed cancer-free). Samples were from 28 males and 34 females (40 < 18 years old, 22 > 18 years old). Clinical staging (Enneking surgical staging system) indicated 29 cases were in stage I-IIA, and 33 cases in stage IIB-IIIA; 27 cases had a tumour diameter of < 6 cm, while 35 cases were ≥ 6 cm; 18 cases presented with distant metastasis and 44 without distant metastasis. Inclusion criteria were as follows: (1) patients who were diagnosed postoperatively as having osteosarcoma by a pathologist; (2) patients who did not receive radiotherapy or chemotherapy before surgery; (3) patients with complete clinical data. Exclusion criteria were as follows: (1) patients with other comorbid malignant tumours; (2) patients with autoimmune diseases; (3) patients with severe liver and kidney dysfunction.

### Cells and main reagent

Osteosarcoma cell lines (SAOS2, MG63 and U2OS) and the human osteoblast cell line (hFOB1.19) were purchased from Procell Co. Ltd (Wuhan, China). PRR11 rabbit anti-human polyclonal antibody (Ab237526), β-catenin rabbit anti-human monoclonal antibody (ab32572), p-β-catenin rabbit anti-human monoclonal antibody (ab75777), GSK-3β mouse anti-human monoclonal antibody (ab93926), p-GSK-3β rabbit anti-human monoclonal antibody (ab68476), c-Myc rabbit anti-human monoclonal antibody (ab32072), CyclinD1 rabbit anti-human monoclonal antibody (ab16663), E-cadherin mouse anti-human monoclonal antibody (Ab1416), Vimentin rabbit anti-human monoclonal antibody (ab92547), Fibronectin rabbit polyclonal antibody (ab2413), total RNA extraction kit were purchased from Abcam (USA). McCoy's 5A and 10% fetal bovine serum-containing DEME were purchased from GIBCO (Thermo Fisher Scientific, USA). Goat anti-mouse IgG, goat anti-rabbit IgG, DAB color kit was purchased from OriGene (USA). The reverse transcription kit HiScript reverse transcriptase and SYBR Green Master Mix were purchased from Novozan Biotechnology Co., Ltd (Nanjing, China). Transwell chamber were purchased from Corning, (USA), and CCK8 kit was purchased from MCE, (USA). PRR11 targeting SiRNA was purchased from Jima (Shanghai, China).

### Methods

#### Immunohistochemical detection of PRR11 expression

The paraffin-embedded sections of the tumor tissues and paracancerous tissues taken are dried, deparaffinized, rehydrated with gradient ethanol, repaired with high temperature and high pressure in citric acid repair solution, and 3% hydrogen peroxide blocks endogenous peroxidase. Add Rabbit anti-human PRR11 (1:800) polyclonal antibody was incubated overnight in a wet box; after taking out and washing, adding goat anti-rabbit IgG antibody, followed by color development, washing, dehydration, transparent mounting, optical microscope photographing observation and analysis. PRR11 present in the tissue stained brown or brownish-yellow in the cytoplasm. A two-tier scoring method combining the positive cell count and staining intensity was used to determine the positive expression of the PRR11 protein. A staining intensity of grade 0 (no staining/-) was assigned 0 points, grade 1 (yellow/+) 1 point, grade 2 (brownish-yellow/++) 2 points, and grade 3 (brown/+++) 3 points. A percentage of positive cells ≤ 5% was assigned 0 points, 6-25% with 1 point, 26-50% with 2 points, 51-75% with 3 points, and > 75% with 4 points. The total score was obtained by multiplying the scores for the percentage of positive cells by the staining intensity. A total score of < 4 points indicated negative or low expression, while ≥ 4 points indicated positive, high expression. Higher scores implied higher PRR11 content. At the same time, the slices were independently determined by experienced pathologists.

#### Cell culture

hFOB1.19 and MG63 cells were cultured with 10% fetal bovine serum-containing DEME, and SAOS2 and U2OS cells were cultured with McCoy's 5A. Place it in an incubator with 5% CO2, 10% humidity and 37 °C for cultivation.

#### Real-time quantitative polymerase chain reaction

Use Trizol reagent to extract total RNA from tissues and cells according to the instructions on the kit, operate according to the instructions of the reverse transcription kit to obtain cDNA, and use the fluorescent quantitative PCR kit to detect the amplification of the target gene. The relative expression of RNA is expressed as 2^-ΔΔCt^, and the experiment is repeated 3 times independently, and the average value is calculated. The primer sequence is shown in Table 1.

#### Total protein extraction and western-blot detection

Use RIPA lysate to extract total protein, and BCA protein assay kit for quantitative detection of protein. Then take 40 μg protein solution per well and load it on SDS-PAGE, transfer it to PVDF membrane, soak the PVDF membrane in the primary antibody incubation solution, and incubate overnight at 4 °C. The next day, wash the membrane with PBS, then incubate with horseradish peroxidase (HRP)-labeled secondary antibody at room temperature for 1 hour, wash off with PBS, and emit ECL light. After taking a picture, use ImageJ software to analyze the relative gray scale of the protein value. Calculation formula: relative expression of target protein = target protein gray value/GAPDH gray value. After the experiment is repeated 3 times, the average value is calculated.

#### U2OS cell transfection

According to the reagent instructions, use LipofectamineTM 2000 cationic lipid transfection reagent for transfection. U2OS cells grown in logarithmic phase were seeded in a cell culture 6-well plate at 5×10^5^ cells/well, and non-specific control SiRNA, PRR11#1, PRR11#2 and PRR11#3 SiRNA were transfected with LipofectamineTM 2000. After 6 hours of transfection, the transfection reagent was aspirated and replaced with normal medium; after 24 hours of culture in a 37 °C, 5% CO2 incubator, it was used for subsequent experiments. Table 2 shows the sequences of the negative control SiRNA-NC and three SiRNA-PRR11.

#### Cell proliferation assay

U2OS cells were transferred and cultured for 24 hours, seeded in 96-well plates and cultured in a 37 °C incubator, and replaced with new culture medium after 24h, 48h, and 72h respectively, added 10 μl CCK-8 solution, and incubated at 37 °C for 1h After that, the microplate reader detects the absorbance at 450 nm, records and draws the proliferation curve. The experiment was repeated three times.

#### Apoptosis detection

Using flow cytometry, after the cells were digested into cell suspension, the cells were resuspended, after rinsing, annexin V (Tianjin Sanjian) was added, and PI dye was added after staining in the dark for 10 minutes, and then detected by flow cytometry. The apoptotic cells are Annexin V positive and PI negative cells, calculate the proportion of apoptotic cells, and the experiment is repeated 3 times.

#### Wound healing assay

Cells are seeded in a six-well plate, and when the cells are covered at the bottom of the six-well plate, use a sterile pipette tip to perpendicular the bottom of the well plate and draw 3 parallel lines along the central axis. After washing with PBS and changing the medium, continue the culture. Take pictures at 0h and 24h after the scratch, and measure the width of the scratch with ImageJ software. The healing rate of the scratch represents the migration capacity. The calculation method is (scratch width of 0h-24h of scratch width)/Scratch width of 0h.

#### Invasion assay

Dilute the Matrigel with DMEM medium at a ratio of 1:8, add 100 μl to the bottom of the Transwell chamber and place the Transwell chamber into a 24-well plate, incubate overnight at 37 °C; observe the number of cells attached to the lower surface of the chamber under a microscope, that is, the number of invaded cells. Invasion rate=number of invaded cells in transfection group/number of invaded cells in control group×100%.

### Statistical analysis

Use SPSS22.0 statistical software to perform statistical analysis on the data. Measurement data were expressed as the mean ± standard deviation (*x*±s). The variance analysis is used to compare the differences between multiple groups, and the t test is used to compare the differences between the two groups. Count data is expressed by rate or component ratio, using χ^2^ test; Kaplan-Meier curve is used to draw the survival curve of patients with osteosarcoma, P<0.05 indicates that the difference is statistically significant.

## Results

### The high expression of PRR11 is related to the clinicopathological characteristics and prognosis of patients with osteosarcoma

**Figure 1:**
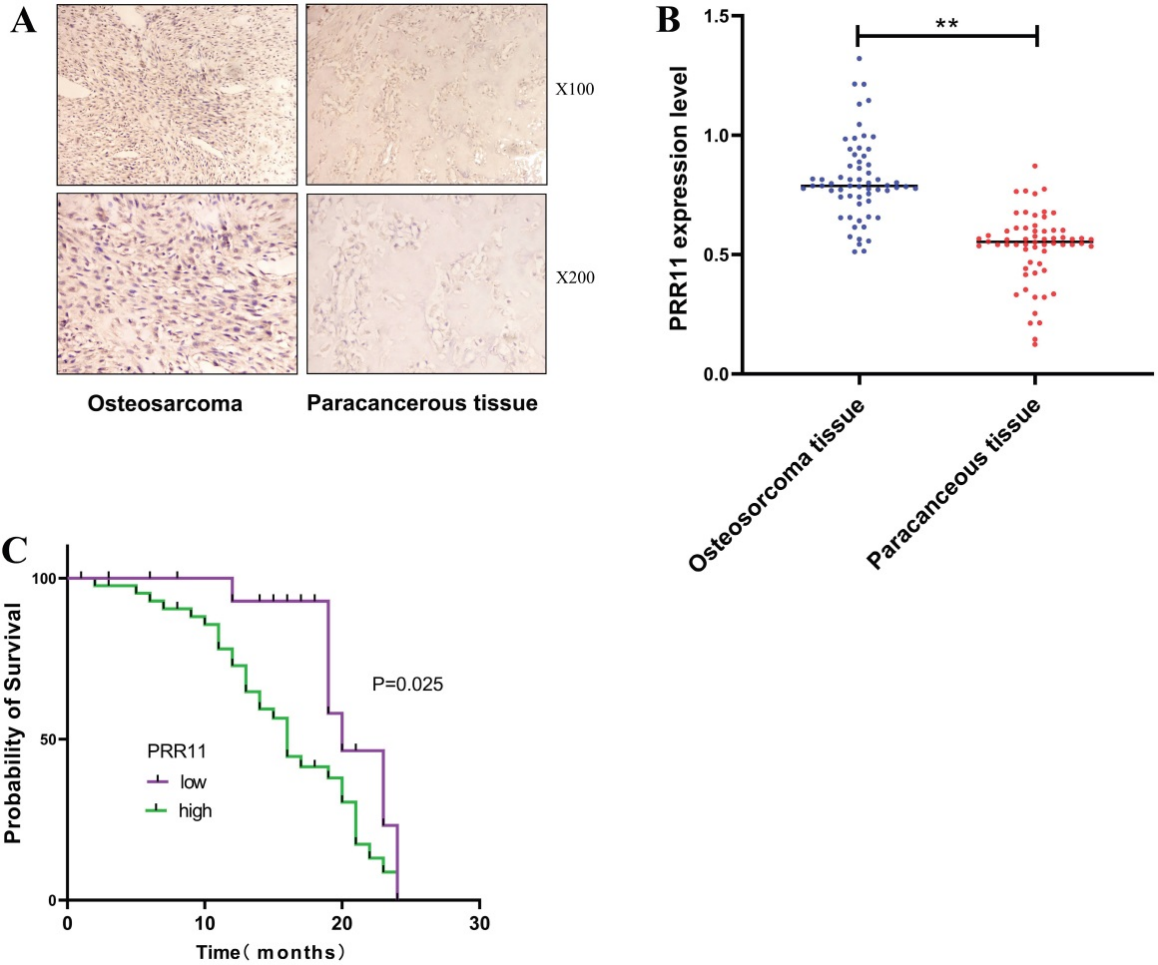
** The high expression of PRR11 is related to the clinicopathological characteristics and prognosis of patients with osteosarcoma. (A)** Expression of PRR11 in human osteosarcoma tissues and paracancerous tissues (magnification: X100 and X200). **(B)** The expression of PRR11 in 62 cases of osteosarcoma tissues and paracancerous tissues. **(C)** Kaplan-Meier analysis of the relationship between the expression of PRR11 and the prognosis of patients with osteosarcoma (*p <0.05; **p <0.01).

IHC detected the expression of PRR11 in 62 cases of osteosarcoma tissues and paracancerous tissues, and found that PRR11 was located in the cytoplasm and presented a brownish-yellow or tan distribution (Figure 1A). According to the staining scoring standard, 44 cases were positive for PRR11 expression (70.09 %). Among the paracancerous tissues, 15 cases were positive for PRR11 (24.19%). Thus, PRR11 protein expression was significantly lower in the paracancerous tissues compared to osteosarcoma tissues (χ² = 27.193, P=0.001, Table 3). The relationship between PRR11 expression in human osteosarcoma tissues and its clinicopathological features were analysed using a χ² test. It was found that the expression level of PRR11 was related to the diameter of the tumor (χ^2^ = 5.514, P=0.019), the clinical stage of the tumor (χ^2^ = 6.598, P=0.010) and lymph node metastasis (χ^2^ = 5.274, P=0.021), but not with gender or age (Table 4). RT-PCR was used to detect the expression of PRR11 mRNA in 62 cases of osteosarcoma tissues and paracancerous tissues. The relative expression of PRR11mRNA in osteosarcoma tissue was 0.79+0.188, which was significantly higher than the relative expression of PRR11mRNA in adjacent tissues of 0.57+0.087 (Figure 1B). Kaplan-Meier was used for survival analysis. 62 patients with osteosarcoma were followed up for 24 months, of which 38 died and 24 survived. The median survival time of 64 patients with osteosarcoma was 19 months. The median survival time of PRR11-positive patients was 16 months, and the median survival time of PRR11-negative patients was 20 months. Plot the survival curve of patients with osteosarcoma from 1 to 24 months of PRR11 positive and negative patients. The survival time of osteosarcoma patients with positive expression of PRR11 protein was significantly lower than that of patients with negative expression of PRR11 (P=0.025, Figure 1C).

### Down-regulating the expression of PRR11 inhibits the proliferation of osteosarcoma cells and promotes apoptosis

**Figure 2:**
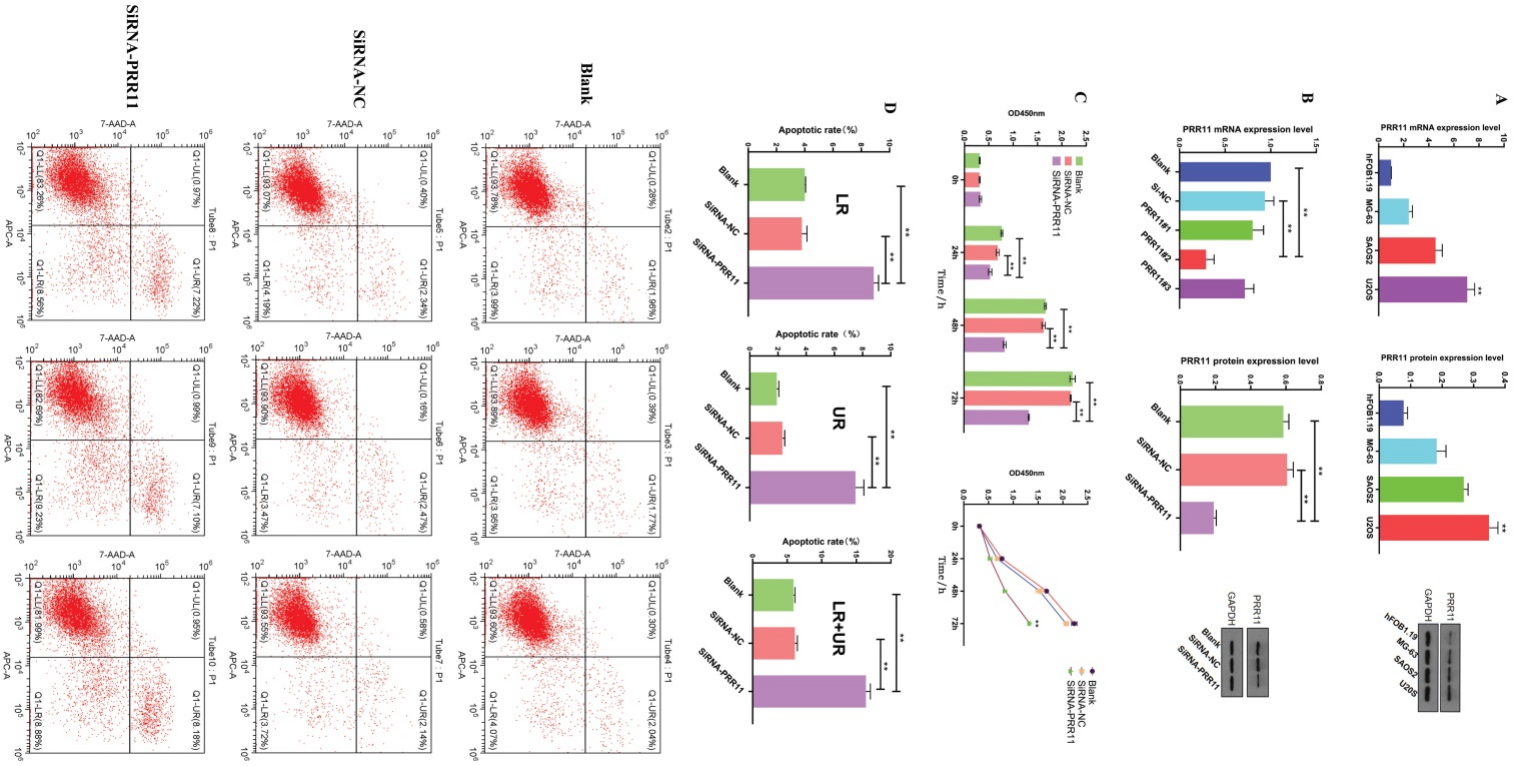
** Down-regulation of PRR11 expression can inhibit the proliferation of osteosarcoma cells and promote apoptosis. (A)** RT-PCR and Western blot analysis of the expression levels of PRR11 osteosarcoma cell lines SAOS2, MG63, U2OS and osteoblast hFoB1.19. **(B)** PRR11 SiRNA and NC were transfected into U2OS cells, and the transfection efficiency was verified by Western blot and RT-PCR. **(C)** The proliferation ability of U2OS cells was measured by CCK-8 at 24 hours, 48 hours and 72 hours after transfection. **(D)** Flow cytometry detected the apoptosis level of U2OS cells after transfection (compared with SiRNA-NC group and Blank group, *p <0.05; **p <0.01).

In order to further study the potential mechanism of PRR11 in osteosarcoma, we selected osteosarcoma cell lines for *in vitro* experiments. First, RT-PCR and Western blotting were used to detect the expression level of PRR11 in the osteosarcoma cell line and the human osteoblast cell line hFOB1.19. Among them, the expression level of PRR11 in U2OS cells was the highest (Figure 2A). Subsequently, we used SiRNA-NC, PRR11#1, PRR11#2 and PRR11#3 SiRNA to transfect U2OS cells respectively, and then used Western blot and qRT-PCR analysis to verify the transfection efficiency. After PRR11#2 SiRNA was transfected into U2OS cells, The expression level of PRR11 was significantly reduced, so PRR11#2 SiRNA with the highest transfection efficiency was selected for subsequent experiments and named SiRNA-PRR11 (Figure 2B). The results of CCK-8 showed that compared with SiRNA-NC group and Blank group, the proliferation ability of U2OS cells was significantly weakened at 24, 48 and 72 h after transfection (Figure 2C). The results of flow cytometry showed that the early apoptosis rate, late apoptosis rate and total apoptosis rate of the cells in the SiRNA-PRR11 group were significantly increased compared with the cells in the SiRNA-NC and Blank groups (Figure 2D).

### Down-regulation of PRR11 inhibits cell migration and invasion

**Figure 3:**
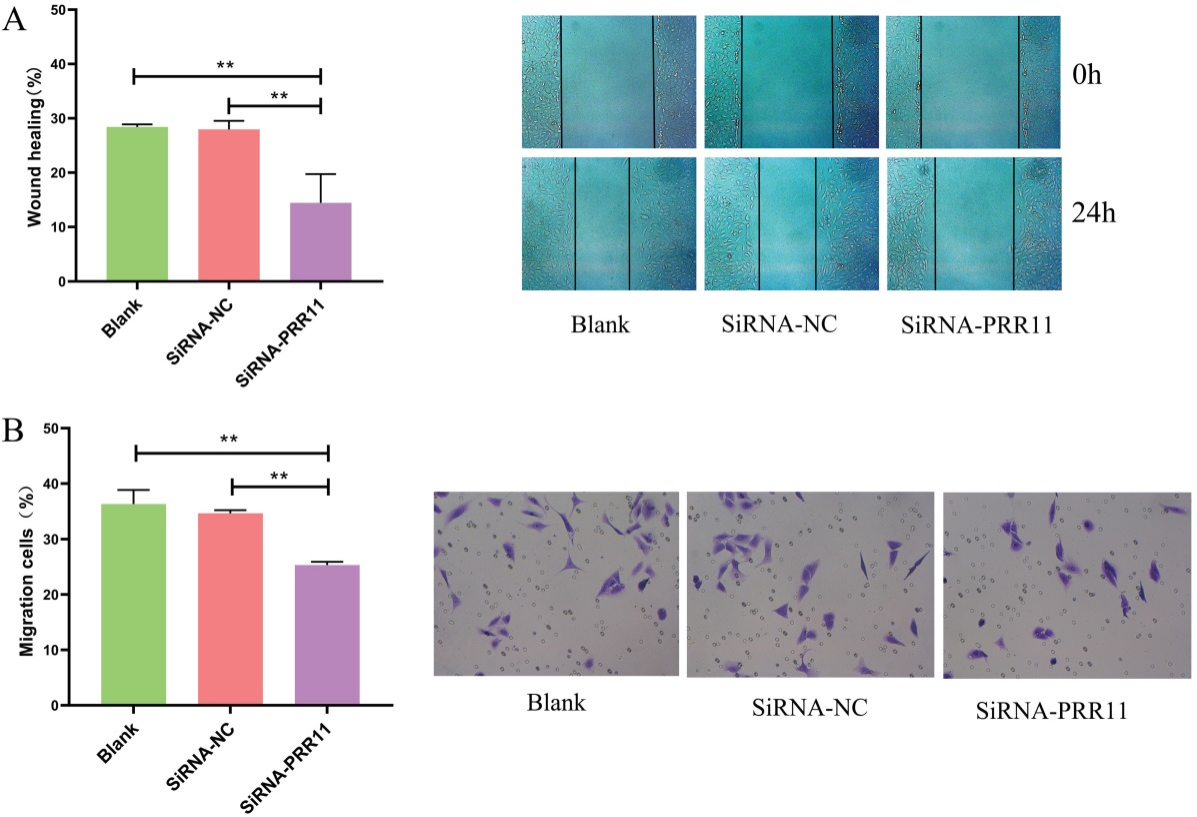
** The effect of down-regulation of PRR11 on the invasion and migration of osteosarcoma U2OS cells. (A)** The scratch test results show that PRR11 targeting SiRNA transfection inhibits the migration of U2OS osteosarcoma cells. **(B)** Transwell results show that PRR11 targeting SiRNA transfection inhibits the invasion of U2OS osteosarcoma cells (compared with SiRNA-NC group and Blank group, *p <0.05; **p <0.01).

Next, we evaluated whether PRR11 can affect the invasion and migration of U2OS cells. The scratch test results showed that U2OS cells had a shorter migration distance than the control group after 24 hours of transfection (Figure 3A). The results of the transwell test showed that U2OS cells invaded less than the control group 24 hours after transfection (Figure 3B).

### Down-regulation of PRR11 affects the expression of Wnt/β-catenin pathway related proteins and EMT related molecules

**Figure 4:**
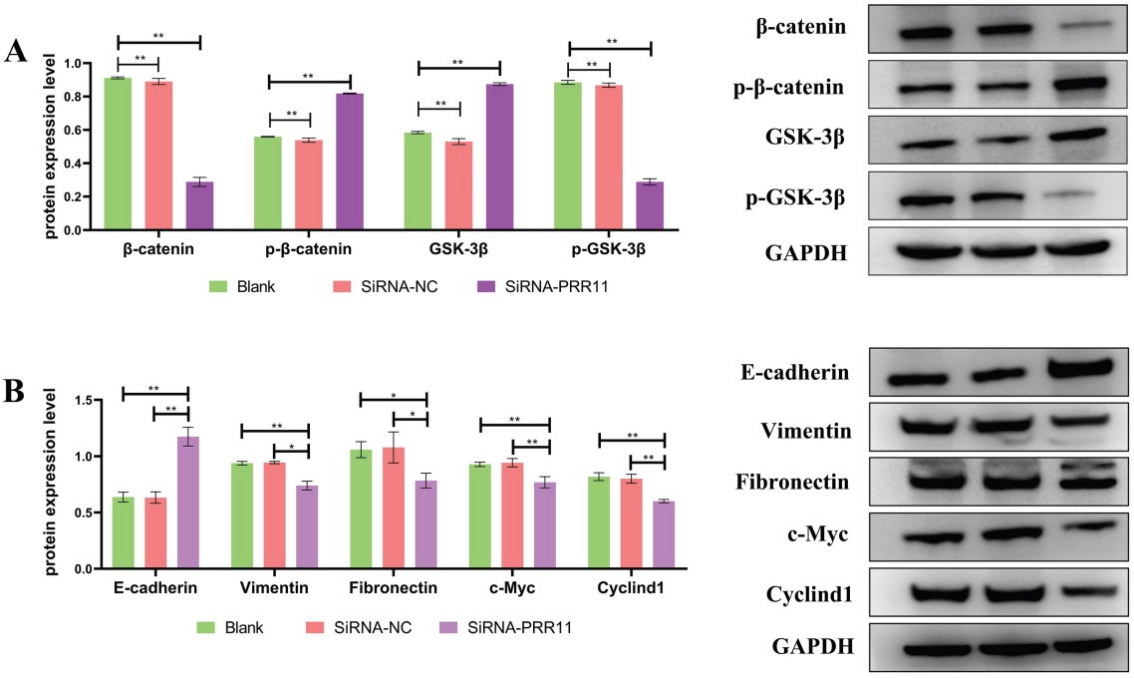
** Down-regulation of PRR11 will affect the expression of Wnt/β-catenin pathway related proteins and EMT related molecules. (A)** After U2OS cell transfection, the expression of Wnt/β-catenin pathway related proteins and downstream regulatory genes. **(B)** After transfection of U2OS cells, the expression of EMT-related molecules (compared with SiRNA-NC group and Blank group, n = 3, *p <0.05; **p <0.01).

Western blotting was used to detect the expression of Wnt/β-catenin pathway related proteins after U2OS cell transfection. It was found that the expression of Wnt/β-catenin pathway related proteins β-catenin and p-GSK-3β decreased, On the contrary, the expression of p-β-catenin and GSK-3β increased (Figure 4A), and the expression of downstream genes cyclinD1 and c-Myc regulated by the Wnt/β-catenin pathway was also lower than that of the control group. Similarly, after U2OS cells were transfected, the expression levels of interstitial markers Vimentin and Fibronectin were significantly lower than those of the control group, and the epithelial marker E-cadherin was significantly higher than that of the control group (Figure 4B).

## Discussion

The tumor-associated gene PRR11 is the first protein-coding gene identified from human full-length cDNA by the Mammal Gene Collection Project of the US National Health Agency in 2000. It is an evolutionarily conserved gene 8. It is located on human chromosome 17q22 and contains 51211 bases. It consists of 9 introns and 10 exons. The translation start codon is located in the second exon, and the stop codon is located in the last exon 9. It has been found that RPS6KB1, BRIP1, TRIM37, PPM1 D and other tumor-related genes are located in this region 10. In the study of normal tissue and tumor tissue microarrays, Ji et al. found that PRR11 gene is differentially expressed. The study confirmed that PRR11 depends on the cell cycle to be expressed in stages. This gene can promote cell proliferation and participate in cell cycle regulation. It may be in the DNA Copy and promote the conversion of S phase to G2/M phase. Recent studies have shown that PRR11 is a key gene that regulates tumorigenesis, and has been regarded as a potential new target for lung cancer diagnosis and treatment in recent years 11. Zhou et al. found that in breast cancer patients, PRR11 is highly expressed by studying breast cancer tissue specimens, and it is related to the prognosis of breast cancer patients 12. Song et al. found that in advanced gastric cancer patients and histological specimens of gastric cancer with a high degree of malignancy, RRR11 showed high expression 13. In esophageal cancer, the 5-year survival rate of high expression of PRR11 was significantly lower than that of the low expression group, which may be related to the interaction of PRR11 with destructive complexes (APC, GSK-3β and AXIN2) to activate the Wnt/β-catenin pathway 14. In our study, it was found that PRR11 is highly expressed in osteosarcoma tissue and is related to tumor size, Enneking stage, and lymph node metastasis. The survival curve results also show that the survival time of patients with positive expression of PRR11 is significantly lower than that of patients with negative expression. This is similar to the results of previous studies of PRR11 in other malignant tumors.

The Wnt/β-catenin signaling pathway, also known as the classic Wnt signaling pathway, is a conservative signaling pathway that participates in various physiological processes, such as proliferation, differentiation, apoptosis, migration and infiltration, and tissue homeostasis 15. The Wnt/β-catenin signaling pathway has been confirmed to play an important role in the occurrence and development of a variety of tumors 16. β-catenin is a key molecule in the Wnt/β-catenin pathway. β-catenin accumulates in the cytoplasm and nucleus, which promotes the enhancement of transcriptional activity of downstream target genes and causes cell proliferation and migration 17. GSK -3β mediates the phosphorylation of β-catenin, promotes its ubiquitination, and is subsequently degraded by the proteasome. GSK-3β is a negative regulator of the Wnt/β-catenin signaling pathway 18, 19. Phosphorylation of GSK-3β to p-GSK-3β can avoid phosphorylation of β-catenin, leading to activation of Wnt/β-catenin signaling pathway, promoting β-catenin to enter the nucleus, and activating downstream target genes such as c-Myc and CyclinD1 transcription, Thereby regulating cell differentiation, growth and migration 20. c-Myc is a downstream effector of β-catenin. It is a proto-oncogene and is widely expressed in human tumors. Its protein is a transcription factor that can affect cell mitosis and participate in the regulation of cell growth and proliferation, cell cycle, and transcriptional differentiation, apoptosis, etc., are closely related to tumor proliferation and apoptosis 21, 22. Studies have shown that the expression of c-Myc is significantly increased in the recurrence and metastasis of osteosarcoma, and its high expression is closely related to the recurrence and metastasis of osteosarcoma 23. Studies have also shown that CyclinD1 is related to the cell cycle progression of a variety of tumors, and inhibiting the level of CyclinD1 is closely related to the reduction of tumorigenicity 24. In our study, we found that PRR11 is highly expressed in osteosarcoma cells. After down-regulating the expression of PRR11 with SiRNA, the proliferation ability of U2OS cells was significantly weakened and the level of apoptosis increased. At the same time, Wnt pathway related proteins p-GSK-3β, β-catenin, protein expression level was significantly reduced, the expression levels of GSK-3β and p-β-catenin protein increased, and the expression levels of downstream genes cyclinD1 and c-Myc decreased. These findings indicate that PRR11 may regulate the proliferation and apoptosis of U2OS cells by regulating the Wnt pathway related proteins and then the downstream expression of CyclinD1 and c-Myc.

Wnt signaling pathway is a key signal transduction pathway that induces EMT in epithelial tissues 25, 26. Recent studies by Liu et al. confirmed that EMT activation can increase the invasion and metastasis ability of osteosarcoma cells, and promote the progression of osteosarcoma 27, 28. The main features of EMT activation include down-regulation of epithelial cell markers such as E-cadherin, and increased expression of mesenchymal phenotypic markers such as Vimentin and N-cadherin 29, 30. Our study found that after PRR11 was down-regulated, the invasion and migration ability of osteosarcoma cells U2OS was significantly reduced. Similarly, the expression levels of interstitial markers Vimentin and Fibronectin in EMT were significantly reduced, and the expression level of epithelial marker E-cadherin was significantly increased. Therefore, we speculate that down-regulation of PRR11 may affect the migration and invasion of osteosarcoma by inhibiting the Wnt /β-catenin signaling pathway and then affecting EMT.

In summary, in this study, we tested the expression of PRR11 in osteosarcoma tissues and osteosarcoma cell lines. It is proved that PRR11 is highly expressed in osteosarcoma tissues and osteosarcoma cells, and the expression level of PRR11 is significantly correlated with the clinicopathological characteristics and prognosis of patients with osteosarcoma. Down-regulating the expression of PRR11 can inhibit the proliferation, migration and invasion of osteosarcoma cells and promote cell apoptosis. At the same time, it will also affect the expression of Wnt pathway related proteins and EMT related proteins. These research data indicate that PRR11 may be involved in the occurrence and development of osteosarcoma as a proto-oncogene, and PRR11 may become a potential indicator and therapeutic target for evaluating the prognosis of osteosarcoma. However, the up-regulation mechanism of PRR11 in osteosarcoma and the molecular mechanism of how to regulate the expression of Wnt pathway and downstream proteins need to be further explored in future research.

## Figures and Tables

**Table 1:** Sequences used for qRT-PCR

Gene	Primer	SequeSiRNA-NCe (5'-3')	PCR Products
Homo GAPDH	Forward	TCAAGAAGGTGGTGAAGCAGG	115bp
	Reverse	TCAAAGGTGGAGGAGTGGGT	
Homo PRR11	Forward	ACCCTCACCAGAAAGAGTCG	215bp
	Reverse	GGTGGCAGATACGAGATGGA	

**Table 2:** SiRNA duplexes used for the knockdown of PRR11

SiRNA	SiRNA duplexes, 5'-3'
SiRNA-NC	UUCUCCGAACGUGUCACGUTT
	ACGUGACACGUUCGGAGAATT
PRR11#1	CAAGUUCAAACAACGAAGATT
	UCUUCGUUGUUUGAACUUGTT
PRR11#2	CCGAGAACUUUACAGUGUATT
	UACACUGUAAAGUUCUCGGTT
PRR11#3	CAUGCAGAUAACAGUUAAATT
	UUUAACUGUUAUCUGCAUGTT

**Table 3:** Differential expression of PRR11 in human osteosarcoma tissues and paired paracancerous tissues

Group	Case	Positive	Negative	Positive rate/%	χ² value	P value
Osteosarcoma tissue	62	44	18	70.09	27.193	0.001
Paracancerous	62	15	47	24.19		

**Table 4:** Relationship of PRR11 positive expression rate and clinicopathological parameters in human osteosarcoma tissues

Parameters	Cases	Postive (n=44)	Negative (n=18)	Postive rate/%	χ² value	P value
**Age (years)**						
<18	40	28	12	70.00	0.051	0.821
≥18	22	16	6	72.73		
**Gender**						
Male	28	20	8	71.43	0.005	0.942
Female	34	24	10	70.59		
**Tumor size/cm**						
≥6 cm	35	29	6	82.86	5.514	0.019*
<6 cm	27	15	12	55.56		
**Enneking stage**						
I-IIA	29	16	13	55.17	6.598	0.010*
IIB-III	33	28	5	84.85		
**Lymph node metastasis**						
Yes	18	17	1	94.44	5.274	0.021*
No	44	27	17	61.36		
